# Supplementary material for: Cdc13 is predominant over Stn1 and Ten1 in preventing chromosome end fusions
Source: eLife. 2020 Aug 5;9:e53144. doi: 10.7554/eLife.53144 (PMC7406354; doi:10.7554/eLife.53144)
Supplement: Supplementary file 1. [file elife-53144-supp1.docx]

| **Supplementary file 1 Mapping primers used in this study** | | |
| --- | --- | --- |
| Primer name | Sequence | Source |
| 1^L^-F | CTCATGTACGTCTCCTCCAAGC | This study |
| 1^L^-R | CTGTCGATGCTGATAGGGCTGT | This study |
| 2^L^-F | CTCCTCAACTGTCGATGATGCC | This study |
| 2^L^-R | GCTAATGCCAGTACCAATGCGA | This study |
| 3^L^-F | TCGCATTGGTACTGGCATTAGC | This study |
| 3^L^-R | TGTCACTGACGGTAGCATGCGA | This study |
| 4^L^-F | CAGAGGGTGTGTCTGCCATGTA | This study |
| 4^L^-R | GGCACTGCGTAGGTAGCAGATT | This study |
| 5^L^-F | CGTCAAGCGTCTTAAGTCGAGGC | This study |
| 5^L^-R | CACGAAGTAGCGCAGGAAACCG | This study |
| 6^L^-F | CCGACCATGACGGAAACGACAA | This study |
| 6^L^-R | GCGGCTGAGCAACGAACAGAAT | This study |
| 7^L^-F | GCGCTTCGAAGTAGAGGAGCC | This study |
| 7^L^-R | GGAGAGGTAGGGTAATGGAGGG | This study |
| 8^L^-F | ACCATCCACCGCCCATCATAAC | This study |
| 8^L^-R | CTGAAGTGGCATCCGTTCAAGC | This study |
| 9^L^-F | GCTCACACACATGTGGGCGCTA | This study |
| 9^L^-R | TCCAGCTGAACAAGTCACCA | This study |
| 10^L^-F | CTGGAGCAGCGGCAACACCG | This study |
| 10^L^-R | TGGCATTCTCACATTCGGCGCA | This study |
| 11^L^-F | CCGTGCCTGTGATACTTCCT | This study |
| 11^L^-R | GCGTATCGAAGAGGAACTGG | This study |
| 12^L^-F | TGACCACATCTTAAACCAACG | This study |
| 12^L^-R | CCCGATATTGACACTGCCGA | This study |
| 13^L^-F | CCAGCGGAAGGTCCATATTGCT | This study |
| 13^L^-R | CAGCCAAATCCACCAGTCTCAG | This study |
| 14^L^-F | GGTGTGATCGCTGCCATCTGTC | This study |
| 14^L^-R | GTGCTACCGACCTGCCGTTTTC | This study |
| 15^L^-F | GTGCGTACGCGAGTTTATCCA | This study |
| 15^L^-R | CGAGCGTGTAATGCTCTGATG | This study |
| 16^L^-F | GAGCCACCAGACGCTAAATA | This study |
| 16^L^-R | CGCTCCACTATCGATGGTTT | This study |
| 17^L^-F | CTCCGACAACGCTGACAGCA | This study |
| 17^L^-R | TTATAGAGCAGCACGGGACC | This study |
| 18^L^-F | TGGTGCCATTGCCGAACCTC | This study |
| 18^L^-R | AAGCAGCGGCAGTGAACTAC | This study |
| 19^L^-F | GATTCTGGAGGTTCAAATAA | This study |
| 19^L^-R | TAATTGCTTATTGATGACCA | This study |
| 20^L^-F | GTACTACGACTACCAGGAAC | This study |
| 20^L^-R | CGCAACTAGCATACATTTAT | This study |
| 21^L^-F | TTCTTGCTGTATTCACGAGC | This study |
| 21^L^-R | TTAGTAGGTCGAGACCAGAA | This study |
| 22^L^-F | TTCAGCAGTAACACGCTGGA | This study |
| 22^L^-R | GCGAATATAGCTTGTAACCA | This study |
| 23^L^-F | TTGCATAGACATCGCTGTCG | This study |
| 23^L^-R | ACCAAATGCACCACTAATCC | This study |
| 24^L^-F | ATCATTCTTCACCTGGTTCT | This study |
| 24^L^-R | TCAATATTTCTGCGCCAGCA | This study |
| 25^L^-F | GGATGAGAGTATCCTGTCTA | This study |
| 25^L^-R | TCATCTGTGGTATTGCAATG | This study |
| 26^L^-F | GAGATTGATGTCATTGACAT | This study |
| 26^L^-R | AGCGAATCTGACCATTGTAT | This study |
| 27^L^-F | CGATTGTGAATTCGGATAGC | This study |
| 27^L^-R | GGTTCAGAAGCTGCAGTGCC | This study |
| 28^L^-F | CTTCTCTGCCTTCAGCCTCT | This study |
| 28^L^-R | GGTATATGTCAACCCATAAG | This study |
| 29^L^-F | CCTTGGCCATGATGGATCAGT | This study |
| 29^L^-R | TAGTAAGCCATGGCTCCAAC | This study |
| 30^L^-F | ATTTACCCTAGAACAACTAG | This study |
| 30^L^-R | TTAATGACCTGAGGCTGCTT | This study |
| 1^R^-R | ACACCACACTACCCTAACAC | This study |
| 1^R^-F | TAGCATCCGTGTGCATATGC | This study |
| 2^R^-R | ATTCCACTCCATCACCCATC | This study |
| 2^R^-F | GGGTGAAGTAAAAGCGTCTG | This study |
| 3^R^-R | GACTTTCTCGTAAGCGTTCC | This study |
| 3^R^-F | GGGACAATTGCGCTTCTTTA | This study |
| 4^R^-R | ATGAGAACGCCCTTCTGGAC | This study |
| 4^R^-F | CGTTATGCCCGAAGTGTATC | This study |
| 5^R^-R | CGGGAGCTACCGTTGAAAAG | This study |
| 5^R^-F | GGAAGTCACTGACGAGGGTT | This study |
| 6^R^-R | CAGCCAGAACCTGTCCGTAAAC | This study |
| 6^R^-F | CGCTCTTGTATCCGGACTGAAC | This study |
| 7^R^-R | GGCTGTGGTTGACCTACCAGAA | This study |
| 7^R^-F | GATGTACGAAGTGAGTGCCCAG | This study |
| 8^R^-R | TATACGGGAGAGTTGCTCTC | This study |
| 8^R^-F | GTTGCTATTGAACCTGGTGT | This study |
| 9^R^-R | CCACAACCTGTCCGCTTGATTC | This study |
| 9^R^-F | GGTAGCAGGCATGAAGGAATCG | This study |
| 10^R^-R | CCCATCTTCATCACCACTCCGT | This study |
| 10^R^-F | CCTCGAAAGGTGCAGGTAATGC | This study |
| 11^R^-R | GCCATCATTGAAGCCGCTCC | This study |
| 11^R^-F | CGCAGAGGCACAATTTAGCA | This study |
| 12^R^-R | GCAGGCTAGCTGCATATTCA | This study |
| 12^R^-F | AGACTGTAACGCCTTGTTGC | This study |
| 13^R^-R | ATGTGTGTTCATGGCTCTTCT | This study |
| 13^R^-F | GTGACTACACCTATGCCTAAG | This study |
| 14^R^-R | CATGGCCGTGCTAGCAGTAACA | This study |
| 14^R^-F | GTCGACTTGTCCTGCCTCATAC | This study |
| 15^R^-R | GTACCGTGCTTAGAACTGGCTC | This study |
| 15^R^-F | CTCTGGAGTGTCCTTTCCCAGT | This study |
| 16^R^-R | CCACCATGGATATTGTGCTG | This study |
| 16^R^-F | CATCCACTTCCCCATAGTGC | This study |
| 17^R^-R | GGCCAACGCCGTATACTAAC | This study |
| 17^R^-F | GCCCTACATGCACAACAAAT | This study |
| 18^R^-R | AATGGAAGACATATCGGCCTACGG | This study |
| 18^R^-F | CAGTAGACACTGGGTCACTTGG | This study |
| 19^R^-R | ATGTTTCTGACATCAGAGCC | This study |
| 19^R^-F | GCGGTAGAAATGGTAGAAGT | This study |
| 20^R^-R | CTAGTGCCTCTGCATCCTCT | This study |
| 20^R^-F | CACTGAAAGTAGACCCGAAG | This study |
| 21^R^-R | CAGTAAAGCAACCACTTCCGCAG | This study |
| 21^R^-F | AGTAGACGACTCTGGAGAGGAAC | This study |
| 22^R^-R | CTGAGCGGACTTCTTCCTTA | This study |
| 22^R^-F | AGCCGAGGAGGCTTTTGGAA | This study |
| 23^R^-R | GGTACCGCTATCGTTGCTGT | This study |
| 23^R^-F | CATTCGTCATCGCCGCATCA | This study |
| 24^R^-R | ACCCGTTCCGACAACGACAA | This study |
| 24^R^-F | GCTACGACTGTTGAAATCGT | This study |
| 25^R^-R | AAGGGCAAACAGGCCTGAGGTA | This study |
| 25^R^-F | AGGTGGAAGAAGCCTCTGTGGT | This study |
| 26^R^-R | CACATCCTGTGAACGGTTACGC | This study |
| 26^R^-F | CTGGCCTATCGGTATCAAGGAC | This study |
| 27^R^-R | CAATCGCAGCAGTACCAGAACC | This study |
| 27^R^-F | GTCTTTAGACCCTTCCGCGGTG | This study |
| 28^R^-R | GGGTGCCAAGGTCATATCGT | This study |
| 28^R^-F | GACAGGCTTCAGGGCAATCT | This study |
| 29^R^-R | TCGCTCTAGGATGACTTTGG | This study |
| 29^R^-F | GCCGCTGCTACTTTCAACTG | This study |
| 30^R^-R | GGCAAGTCCTGTTCTGTGTGGT | This study |
| 30^R^-F | CGGTTCTAACCTTACCGTCCAC | This study |
| 31^R^-R | CTGGTGGTCGTAACTTGGGTCG | This study |
| 31^R^-F | AAGATCTCGGCGTCGGTTCTGA | This study |
| 32^R^-R | CCCTAGTGACCAGCTTGGATGT | This study |
| 32^R^-F | GGGCACAGACATCTGTACTTCTG | This study |
| 33^R^-R | CCACCTTCCACTAAGGACGTAGA | This study |
| 33^R^-F | CGTCACCGTTGGTCCAGAACGA | This study |
| 34^R^-R | TCGCGTGCTTATTCTCAGGAGC | This study |
| 34^R^-F | TGAGCGTGCAGTAGCAGGTGTT | This study |
| 35^R^-R | AGGGTCTACGCGGTCCATAATC | This study |
| 35^R^-F | GACCTGCCTTGCTACCGTCTAT | This study |
