# Supplementary material for: Cdc13 is predominant over Stn1 and Ten1 in preventing chromosome end fusions
Source: eLife. 2020 Aug 5;9:e53144. doi: 10.7554/eLife.53144 (PMC7406354; doi:10.7554/eLife.53144)
Supplement: Supplementary file 2. [file elife-53144-supp2.docx]

| **Supplementary file 2 Yeast strains used in this study** | | |
| --- | --- | --- |
| Strain | Genotype | Source |
| BY4742 | *MATα his3*Δ*1 leu2*Δ*0 lys2*Δ*0 ura3*Δ*0* | Euroscarf |
| SY14 | *MATα his3*Δ*1 leu2*Δ*0 lys2*Δ*0 ura3*Δ*0* | (Shao et al., 2018) |
| SY15 | *MATα his3*Δ*1 leu2*Δ*0 lys2*Δ*0 ura3*Δ*0* | (Shao et al., 2019) |
| SY14/SY14^a^ | *MATα/a* *his3*Δ*1 leu2*Δ*0 lys2*Δ*0 ura3*Δ*0* | (Shao et al., 2018) |
| WZJ0001 | SY14/SY14^a^ *RAP1/rap1*Δ::*HIS3/CEN* pRS316-*RAP1* | This study |
| WZJ0002 | SY14/SY14^a^  *CDC13/cdc13*Δ::*HIS3/CEN* pRS316-*CDC13* | This study |
| WZJ0003 | SY14/SY14^a^ *STN1/stn1*Δ::*HIS3/CEN* pRS316-*STN1* | This study |
| WZJ0004 | SY14/SY14^a^ *TEN1/ten1*Δ::*HIS3/CEN* pRS316-*TEN1* | This study |
| WZJ0005 | SY15 *rap1*Δ::*HIS3/CEN* pRS316-*RAP1* | This study |
| WZJ0006 | SY15 *cdc13*Δ::*HIS3/CEN* pRS316-*CDC13* | This study |
| WZJ0007 | SY15 *stn1*Δ::*HIS3/CEN* pRS316-*STN1* | This study |
| WZJ0008 | SY15 *ten1*Δ::*HIS3/CEN* pRS316-*TEN1* | This study |
| WZJ0009 | SY15 *RAP1/CEN* pRS316-*RAP1* | This study |
| WZJ0010 | SY15 *CDC13/CEN* pRS316-*CDC13* | This study |
| WZJ0011 | SY15 *STN1/CEN* pRS316-*STN1* | This study |
| WZJ0012 | SY15 *TEN1/CEN* pRS316-*TEN1* | This study |
| WZJ0013 | SY15 *cdc13*Δ::*HIS3 X-R*::*KanMX-CA255/CEN* pRS316-*CDC13* | This study |
| WZJ0014 | SY15 *stn1*Δ::*HIS3 X-R*::*KanMX-CA255/CEN* pRS316-*STN1* | This study |
| WZJ0015 | SY15 *ten1*Δ::*HIS3 X-R*::*KanMX-CA255/CEN* pRS316-*TEN1* | This study |
| WZJ0016 | SY15 *cdc13*Δ::*HIS3 stn1*Δ::*LEU2 X-R*::*KanMX-CA255/CEN* pRS316*-CDC13-STN1* | This study |
| WZJ0017 | SY15 *cdc13*Δ::*HIS3 ten1*Δ::*LEU2 X-R*::*KanMX-CA255/CEN* pRS316*-CDC13-TEN1* | This study |
| WZJ0018 | SY15 *stn1*Δ::*HIS3 ten1*Δ::*LEU2 X-R*::*KanMX-CA255/CEN* pRS316*-STN1-TEN1* | This study |
| WZJ0019 | SY15 *cdc13*Δ::*HIS3 X-R*::*KanMX-TG255/CEN* pRS316-*CDC13* | This study |
| WZJ0020 | SY15 *stn1*Δ::*HIS3 X-R*::*KanMX-TG255/CEN* pRS316-*STN1* | This study |
| WZJ0021 | SY15 *ten1*Δ::*HIS3 X-R*::*KanMX-TG255/CEN* pRS316-*TEN1* | This study |
| WZJ0022 | SY15 *cdc13*Δ::*HIS3 stn1*Δ::*LEU2 X-R*::*KanMX-TG255/CEN* pRS316*-CDC13-STN1* | This study |
| WZJ0023 | SY15 *cdc13*Δ::*HIS3 ten1*Δ::*LEU2 X-R*::*KanMX-TG255/CEN* pRS316*-CDC13-TEN1* | This study |
| WZJ0024 | SY15 *stn1*Δ::*HIS3 ten1*Δ::*LEU2 X-R*::*KanMX-TG255/CEN* pRS316*-STN1-TEN1* | This study |
| WZJ0025 | SY14 *cdc13*Δ::*HIS3 yku70*Δ::*LEU2/CEN* pRS316-*CDC13* | This study |
| WZJ0026 | SY14 *cdc13*Δ::*HIS3 rad52*Δ::*LEU2/CEN* pRS316-*CDC13* | This study |
| WZJ0027 | SY14 *yku70*Δ::*LEU2* | This study |
| WZJ0028 | SY14 *lig4*Δ::*HIS3* | This study |
| WZJ0029 | SY14 *cdc13*Δ::*HIS3 pol32*Δ::*LEU2/CEN* pRS316-*CDC13* | This study |
| WZJ0030 | SY14/SY14^a^ *cdc13*Δ::*HIS3 stn1*Δ::*LEU2/CEN* pRS316*-CDC13-STN1* | This study |
| WZJ0031 | SY14/SY14^a^ *cdc13*Δ::*HIS3 ten1*Δ::*LEU2/CEN* pRS316*-CDC13-TEN1* | This study |
| WZJ0032 | SY14/SY14^a^ *stn1*Δ::*HIS3 ten1*Δ::*LEU2/CEN* pRS316*-STN1-TEN1* | This study |
| WZJ0033 | SY14 *cdc13*Δ::*HIS3 X-R*::*KanMX-CA255/CEN* pRS316-*CDC13* | This study |
| WZJ0034 | SY14 *stn1*Δ::*HIS3 X-R*::*KanMX-CA255/CEN* pRS316-*STN1* | This study |
| WZJ0035 | SY14 *ten1*Δ::*HIS3 X-R*::*KanMX-CA255/CEN* pRS316-*TEN1* | This study |
| WZJ0036 | SY14 *cdc13*Δ::*HIS3 stn1*Δ::*LEU2 X-R*::*KanMX-CA255/CEN* pRS316*-CDC13-STN1* | This study |
| WZJ0037 | SY14 *cdc13*Δ::*HIS3 ten1*Δ::*LEU2 X-R*::*KanMX-CA255/CEN* pRS316*-CDC13-TEN1* | This study |
| WZJ0038 | SY14 *stn1*Δ::*HIS3 ten1*Δ::*LEU2 X-R*::*KanMX-CA255/CEN* pRS316*-STN1-TEN1* | This study |
| WZJ0039 | SY14 *tlc1*Δ::*LEU2/CEN* pRS316-*TLC1* | This study |
| WZJ0040 | SY15 *tlc1*Δ::*LEU2/CEN* pRS316-*TLC1* | This study |
| WZJ0041 | SY14 *tlc1*Δ::*LEU2 rad52Δ::HIS3 /CEN* pRS316-*TLC1* | This study |
| WZJ0042 | SY14 *tlc1*Δ::*LEU2* *yku70*Δ::*HIS3 /CEN* pRS316-*TLC1* | This study |
| WZJ0043 | SY14 *tlc1*Δ::*LEU2* *pol32*Δ::*HIS3 /CEN* pRS316-*TLC1* | This study |

**References**

Shao Y, Lu N, Cai C, Zhou F, Wang S, Zhao Z, Zhao G, Zhou J-Q, Xue X, Qin Z (2019) A single circular chromosome yeast. *Cell research* 29: 87-89

Shao Y, Lu N, Wu Z, Cai C, Wang S, Zhang LL, Zhou F, Xiao S, Liu L, Zeng X, Zheng H, Yang C, Zhao Z, Zhao G, Zhou JQ, Xue X, Qin Z (2018) Creating a functional single-chromosome yeast. *Nature* 560: 331-335
